# Investigation on the Anti-Cancer Effects of HER2-Targeted CAR-T Cells Engineered Using the *PiggyBac* Transposon System

**DOI:** 10.32604/or.2025.065394

**Published:** 2025-10-22

**Authors:** Tian-Tian Li, Ming-Yao Meng, Zheng Yu, Yang-Fan Guo, Yi-Yi Zhao, Hui Gao, Li-Li Yang, Li-Rong Yang, Meng-Yuan Chu, Shan He, Yuan Liu, Xiao-Dan Wang, Wen-Ju Wang, Zong-Liu Hou, Li-Wei Liao, Lin Li

**Affiliations:** 1 Central Laboratory, Yan’an Hospital Affiliated to Kunming Medical University, Kunming, 650051, China; 2 Key Laboratory of Tumor Immunological Prevention and Treatment of Yunnan Province, Yan’an Hospital Affiliated to Kunming Medical University, Kunming, 650051, China; 3 Graduate School, Kunming Medical University, Kunming, 650050, China; 4 Yunnan Cell Biology and Clinical Translation Research Center, Yan’an Hospital Affiliated to Kunming Medical University, Kunming, 650051, China; 5 School of Life Science and Technology, ShanghaiTech University, Shanghai, 201210, China

**Keywords:** Chimeric antigen receptor T, human endothelial growth factor receptor 2, cell therapy, *PiggyBac* transposase

## Abstract

**Background:**

Chimeric antigen receptor T (CAR-T) cell therapies have demonstrated significant clinical efficacy in hematological malignancies. However, their application to solid tumors remains substantially limited by multiple challenges, including the risk of off-target effects. Hence, optimizing CAR-T cells for stronger antigen binding is essential.

**Methods:**

In this study, we employed a classical anti-human endothelial growth factor receptor 2 (HER2) single-chain variable fragment (scFv) derived from trastuzumab, alongside an anti-HER2-13 scFv identified from a combinatorial cellular CAR library, for the construction of a third-generation CAR-T cell. Meanwhile, the phenotypes and both *in vitro* and *in vivo* functions of CAR-T cells transduced with the two scFvs via *PiggyBac* transposon-mediated gene transfer were compared.

**Results:**

The optimal ratio between the *PiggyBac* HER2-CAR-puro transposon and the Super *PiggyBac* transposase plasmid differed during the construction of the two HER2-targeted CAR-T cell types. The expansion abilities, CD3^+^CAR^+^ population, CD4^+^CAR^+^/CD8^+^CAR^+^ proportions, and memory and exhaustion markers between the two CAR-T groups were similar after using the optimized proportion of plasmid. Both CAR-T cell types exhibited significant antitumor activity, with the anti-HER2-13 CAR-T cells demonstrating superior target specificity. Therapeutic effects were observed with both CAR-T cells and trastuzumab in the MDA-MB-231^HER2+^ breast tumor xenograft model, with anti-HER2-13 CAR-T cells demonstrating slightly enhanced efficacy and no evident off-target toxicity.

**Conclusion:**

These results highlight the potential of anti-HER2-13 CAR-T cells to serve as a safer and more efficacious alternative in HER2-targeted therapy.

## 1 Introduction

Human endothelial growth factor receptor 2 (HER2), a member of the EGFR family, is a leucine-rich transmembrane kinase receptor closely related to cell growth and differentiation [1,2]. Overexpression of HER2 usually induces tyrosine kinase domain activation, sustaining oncogenic signaling cascades that promote aberrant epithelial cell growth, proliferation and differentiation, disrupting normal physiological processes and facilitating malignant transformation [3]. It has been shown that overexpression of HER2 is associated with the development and progression of cancers such as breast cancer, prostate cancer, and gastric cancer [4–6]. At present, many anti-HER2 monoclonal antibody drugs have been approved by the FDA and have shown good therapeutic effects, particularly trastuzumab, which has been shown to improve progression-free survival and overall survival in patients harboring HER2-positive malignancies. However, as a targeted therapy, trastuzumab’s clinical efficacy is significantly limited by the development of drug resistance [7–9].

Chimeric antigen receptor T-cell therapy (CAR-T) has achieved remarkable clinical success in treating hematological cancers. CAR-T cells are generated through genetic modification of autologous or allogeneic peripheral blood-derived T cells to express synthetic chimeric antigen receptors targeting tumor-associated antigens, independent of major soluble complex (MHC) presentation, so that to can recognize and target cancer cells for killing [10,11]. Our previous studies developed a third-generation CAR structure incorporating a human CD19-specific scFv, linked via a CD28 transmembrane domain and a CD8 hinge to the intracellular signaling domains of CD28, 4-1BB, and CD3ζ [12,13]. To further investigate the effects of our CAR-T cells on solid tumors, we constructed the anti-HER2 CAR-T cells with the above third generation of CAR, while containing a classical humanized monoclonal antibody (mAb) trastuzumab-derived scFv (Supplementary Table S1). Nevertheless, one clinical study demonstrated that the CAR based on trastuzumab might cause serious adverse events [2]. Therefore, in order to reduce the off-target effect, a novel sequence of humanized mAb against HER2 called anti-HER2-13 scFv (Supplementary Table S1) was screened from the combinatorial cellular library [14]. In this study, a third-generation CAR was also constructed using a screened novel humanized anti-HER2-13 scFv, and a comparative analysis was performed to evaluate the specific cytotoxicity, proliferation and phenotypic features of anti-HER2 and anti-HER2-13 CAR-T cells against HER2-positive malignant cells in culture systems and xenograft model under standardized culture conditions.

Moreover, despite the FDA approval of six CAR-T cell therapies for hematologic malignancies [15–18], the viral vector-based approach commonly used for CAR construction presents significant limitations including substantial manufacturing expenses, prolonged production timelines, and potential risks such as contamination with infectious agents and severe post-treatment toxicities [19,20]. Hence, the *PiggyBac* transposon/transposase system (PB), a highly efficient non-viral vector with site-specific integration potential, was used in our present study. While our previous research had found that anti-CD19 CAR-T cells could be effectively transfected via the *PiggyBac* transposon system, with CAR expression level surpassing 40% [12], some studies have suggested that transfection efficiency is substantially affected by the ratio of transposons to transposases plasmids [21,22]. In view of this, further optimization of the ratio between the transposons and transposases plasmids needs to be carried out in this study to improve the transfection efficiency of anti-HER2 and anti-HER2-13 CAR-T cells. Here, HER2-specific CAR-T cells were generated using a novel scFv (anti-HER2-13) through a non-viral *PiggyBac* transposon system, resulting in stable and robust CAR expression and enhanced specificity toward HER2-positive tumor cells *in vitro* and *in vivo*. Our findings reveal that anti-HER2-13 CAR-T could be a potential treatment avenue in HER2-positive cancer.

## 2 Materials and Methods

### 2.1 Cell Lines and Cell Culture

The different cancer cell lines including SPC-A-1 (lung adenocarcinoma), MDA-MB-231 and SKBR-3 (breast cancer), which were obtained from the Cell Bank of Shanghai Institutes for Biological Sciences, Chinese Academy of Sciences (Shanghai, China) and Kunming Cell Bank of Type Culture Collection (Kunming Institute of Zoology, Kunming, China), were maintained in RPMI1640 medium (Biological Industries, Cat#01-100-1ACS, Israel) containing 10% FBS (Biological Industries, Cat#04-001-1ACS, Israel) and 1% penicillin/streptomycin (Biological Industries, Cat#03-031-1B, Israel) at 37°C in a humidified 5% CO_2_ atmosphere. Mycoplasma contamination in cell cultures was detected by Nested PCR using the Mycoplasma PCR Detection Kit (Beyotime Biotechnology, Cat#C0301S, Shanghai, China) following the supplier’s recommended protocol.

Peripheral blood mononuclear cells (PBMCs) were harvested from the peripheral blood samples from healthy volunteers, all of whom tested negative for HBV, HCV, HIV, and syphilis in pre-transfusion infectious disease screening. An ethics committee of Kunming Medical University, Yan’an Affiliated Hospital, approved this study (2017-074-01). We hereby declare that informed consent was obtained for the use of human samples in the experiment and that the privacy of the donors was always respected.

### 2.2 CAR Vector Construction and HADDOCK Prediction

**Figure 1:**
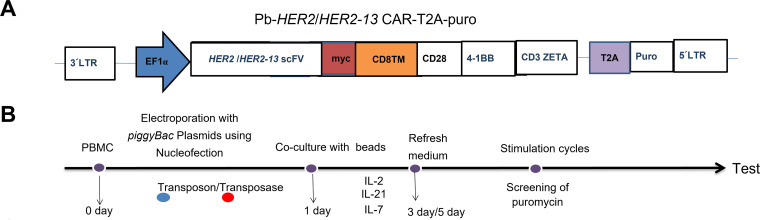

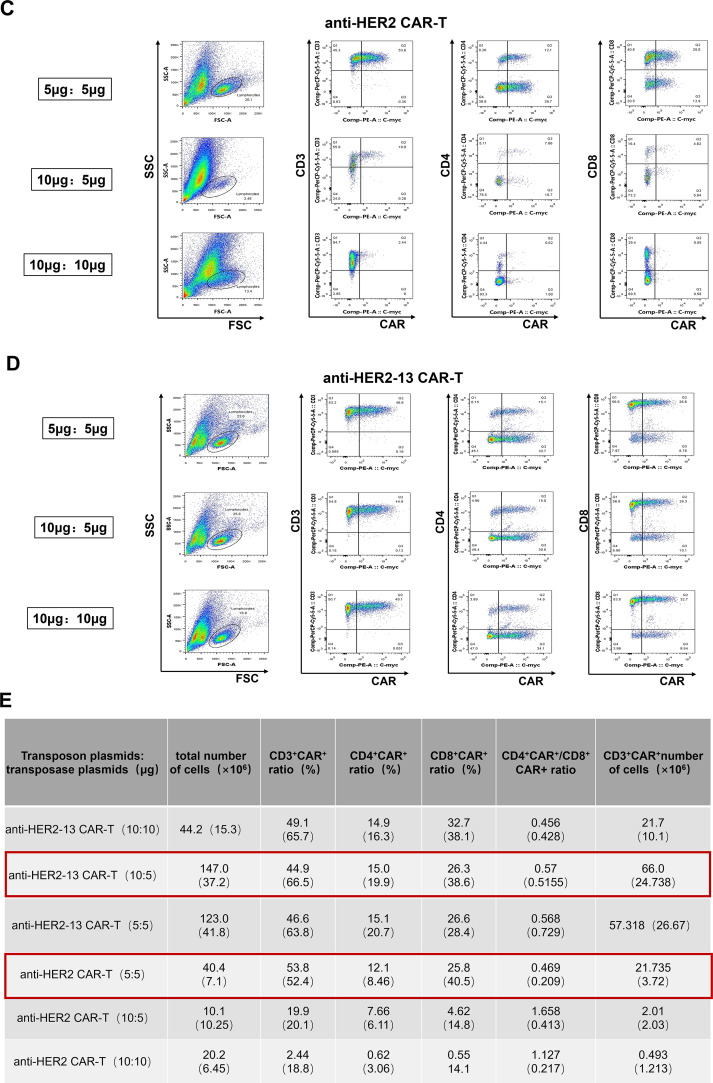
The optimization of the ratio of transposon plasmid and transposase plasmid for transfection of anti-HER2 and anti-HER2-13 CAR-T cells. (**A**) The schematic representation of the anti-HER2/HER2-13 CAR structure. (**B**) Diagram of the construction and expansion of CAR-T cells. On day 0, the isolated PBMC were electroporated with *PiggyBac* transposase plasmid along with transposon DNA plasmids encoding the PB-HER2(13)-CAR-puro. On day 1, magnetic beads and IL-2, IL-7 and IL-21 were added for expansion for 3–5 days, then the cells were followed by removal of the magnetic beads and continued culture for approximately 18–21 days. (**C**,**D**) The representative flow cytometry graphs of CAR^+^ expression in CAR-T conducted with three different ratios (μg:μg) of transposon plasmid and transposase plasmid. (**E**) The analytic results of the total cell proliferation, CD3^+^CAR^+^, CD4^+^CAR^+^, CD8^+^CAR^+^, CD4^+^CAR^+^/CD8^+^CAR^+^ and CD3^+^CAR^+^ cell numbers. The data was from two different donors

The *PiggyBac* DNA transposon and transposase system (PB) was employed to mediate gene transfer into donors’ freshly isolated T lymphocytes, thereby inducing the expression of human HER2-specific CAR. The CAR consists of a scFv targeting human HER2 (either anti-HER2 or anti-HER2-13) and is incorporated CD28, 4-1BB and CD3ζ intracellular signaling domains, connected through a CD28-derived transmembrane region and CD8α hinge domain (Fig. 1A). The cDNA encoding the HER2/HER2-13-specific chimeric antigen receptor (CAR) was custom synthesized by GenScript (Piscataway, NJ, USA). T2A and the puromycin resistance (PuroR) coding sequence were excised from pCDH-EF1-MCS-T2A-Puro lentiviral vector (System Bioscience, Catalog Number CD527A-1, San Francisco, CA, USA) using digestion, and anti-HER2/HER2-13 scFv coding sequence was directionally cloned into the PB-EF1-MCS-IRES-Neo *piggyBac* vector (System Biosciences, Catalog Number PB533A-2), restricted using Xba1 and Bgl2. The designed sites collectively replaced the MCS-IRES-neo fragment in the vector backbone.

The structure of the human HER2 antigen was first predicted using AlphaFold3. Subsequently, potential binding regions between the antigen and the two constructed scFv fragments were predicted using BepiPred-3.0 (https://services.healthtech.dtu.dk/services/BepiPred-3.0/ (accessed on 01 August 2025)). Finally, HADDOCK (High Ambiguity Driven Docking)-based molecular docking was employed to calculate the binding score between the antigen and humanized anti-HER2-13 or anti-HER2 scFv [23,24].

### 2.3 HER2-Positive Cells Transfection and CAR-T Cell Transduction

HER2 expression was knocked down (or overexpressed) in SKBR-3, MDA-MB-231, and SPC-A-1 cell lines through lentiviral vector-mediated transduction. A lentivirus-based shRNA expression vector designed to target HER2 was constructed by Shanghai Genechem Co., Ltd. (Shanghai, China). The gene-silencing oligonucleotide sequence specifically targeting the HER2 cDNA is shHER2-111366: GCAGTTACCAGTGCCAATATC; shHER2-111367: GCTCCAAGTGTTTGAGACTCT; shHER2-111368: GAACACAGCGGTGTGAGAAGT, and a non-targeting scrambled shRNA (shNC) served as the experimental control: TTCTCCGAACGTGTCACGT. SKBR-3 cells were transfected with each lentiviral construct at an optimal multiplicity of infection (MOI) of 50. Transfection efficiency was assessed by monitoring GFP-positive cells under a fluorescence microscope (Bio-Rad, 742BR1307, Hercules, CA, USA). The HER2 overexpression procedure in SPC-A-1 and MDA-MB-231 cells followed the same protocol as previously described.

Anti-HER2/HER2-13 CAR-T cells were transfected with PB using 2b-Nucleofector kit (Lonza, VVPA-1002, Allendale, NJ, USA) by an electroporator (Nucleofector 2b, 91720139, Lonza). The procedure was according to our previous studies, with minor modifications [12,13]. Briefly, peripheral blood-derived PBMC were transfected with either the PB-HER2 or HER2-13 CAR-puro plasmid in combination with the Super *PiggyBac* transposase. On the first day after electroporation, CD3/CD28 magnetic beads (ThermoFisher, 11131D, Waltham, USA, USA) was added to cells for stimulation at a ratio of 2 beads per cell, and the cytokines IL-2 (Peprotech, AF-200-07-250UG, Rocky Hill, NJ, USA) (300 U/mL), IL-7 (Peprotech, AF-200-02-1MG, USA) (10 ng/mL) and IL-21 (Peprotech, AF-200-21-1MG, USA) (30 ng/mL) purchased from Peprotech, New Jersey, USA, were also added into X-vivo 15 culture system (Lonza, 04-418, USA) at the same time. On the fifth day, the beads were removed from the CAR-T cells culture system. CAR-T cells were selected and enriched by applying gradient concentrations of puromycin in culture medium starting on day 12 post-transfection. The cytokine-containing medium was refreshed at 48 h intervals for a duration of 18–20 days (Fig. 1B).

### 2.4 Cryopreservation and Thawing

CAR-T cells products underwent cryopreservation in NutriFreez® D10 Cryopreservation Medium (Biological Industries, 05-713-1A, Israel) at 2 × 10^7^ cells/mL in 1.8 mL chilled cryo-vials (Thermo Scientific, 377267, Lyngby, Denmark). The cryopreserved vials were placed in a Nalgene Cryo 1°C Freezing Container and preserved in a −80°C refrigerator. The cryopreserved vials were placed in long-term liquid nitrogen storage.

After one month or six months in liquid nitrogen, cryopreserved cells were rapidly thawed in a 37°C water bath with gentle agitation until minimal ice crystallization persisted, then gently resuspended in 3 mL of X-vivo 15 medium (pre-equilibrated to 37°C) within a 15 mL tube, pelleted by centrifugation (1000 rpm, 5 min) (Thermo, SN09060479, Waltham, MA, USA) for 5 min and washed with X-vivo 15 medium for living cell counting and further flow cytometry analysis.

### 2.5 Flow Cytometry Detection

CAR-T cell phenotypes were profiled by flow cytometry after the cells *ex vivo* expansion or thawing. In brief, 5 × 10^6^ cells were collected from each group of CAR-T cells, and cell pellets were obtained by centrifugation at 1000 rpm for 5 min, followed by careful aspiration of the supernatant, and 100 μL sheath fluid was used to resuspend the cells. Subsequently, the cell suspension was slightly mixed and incubated with the antibody at 4°C for 20 min in the dark. After incubation, cellular immunophenotyping was performed using flow cytometric analysis. The following antibodies were used in flow cytometry detection: anti-CD3 PerCP-Cy5.5 (BD Pharmingen, Clone SP34-2, San Jose, CA, USA), anti-CD4 PerCP-Cy5.5 (BD Pharmingen, Clone L200), anti-CD8 PerCP-Cy5.5 (BD Pharmingen, Clone RPA-T8), anti-Myc-Tag PE (CST, #3739), anti-CD44 PerCP-Cy5.5 (BD Pharmingen, Clone G44-26), anti-CD62L PE (BD Pharmingen), anti-Tim-3 PE (BD Pharmingen, Clone 7D3), anti-PD-1 PE (BD Pharmingen), anti-LAG-3 PE (BD Pharmingen, Clone T47-530). Antibodies were used at 1:51 dilutions (prepared by adding 1 μL antibody to 50 μL flow buffer supplemented with 1% BSA). The data were obtained from a BD FACSCanto and were analyzed using FlowJo version 10.5.3 (BD Biosciences).

### 2.6 Western Blot

Overexpression and knockdown of HER2 in tumor cells were determined by western blot. Following PBS washing (4°C), cells were lysed using ice-cold RIPA buffer (Beyotime Biotechnology, P0013, Shanghai, China) containing protease inhibitor mixtures (Roche Molecular Biochemicals, 4906845001, Basel, Switzerland). Protein content was assessed via the BCA Protein Concentration Assay (Beyotime Biotechnology, P0010), and 30 μg of total protein was loaded and electrophoresed on 10% SDS-PAGE gels, which were then transferred to a hydrophobic PVDF (Polyvinylidene Fluoride) membrane (Millipore, Carrigtwohill, ISEQ00010, Ireland). The membrane was blocked with 5% (w/v) non-fat dry milk in Tris-buffered saline + 0.1% Tween-20 (TBST, 9005-64-5, Sigma-Aldrich, USA) for about 1–2 h at room temperature. They were then incubated overnight at 4°C with monoclonal rabbit anti-human HER2 antibody (Cell Signaling Technology, 4290S, Danvers, MA, USA, 1:1000) or anti-β-actin mAb (mouse origin) (Proteintech, 66009-1, Rosemont, IL, USA, 1:1000). The membrane was then washed with TBST and incubated with the secondary HRP-goat anti-mouse IgG (H + L) (Proteintech, RGAM001, USA) or HRP-goat anti-rabbit IgG (H + L) (Proteintech, 10435-1-AP, USA) at room temperature for 1 h, with both antibodies at a dilution of 1:5000. Protein expression was visualized using ECL protein blotting substrate (Proteintech, PK10003, USA) and images were captured by using a BIO-RAD instrument (Bio-Rad, 732BR1872, USA).

### 2.7 Cytotoxicity Assays

The water-soluble tetrazolium salt (WST-8)-based CCK-8 assay (CCK-8, CK04-500T, DOJINDO, Kumamoto, Japan) was employed to quantify the tumoricidal effects of CAR-T cells. Briefly, tumor cells (5000 cells/each well) were seeded into 96-well plates overnight, then co-cultured with serially diluted CAR-T cells to quantify cytotoxicity over a range of E:T (effector cell/target cell) ratios. After 24 h, following removal of supernatant and non-adherent cells, the viability of residual tumor cells was quantified using CCK-8 reagent in accordance with the manufacturer’s instructions after washing the plate twice with PBS. Three replicate wells were set up for each experiment. The experiments were conducted in triplicate with three independently produced CAR-T cell batches.

### 2.8 Cytokines Detection

Non-treated T cells (NT)/CAR-T cells were co-incubated with tumor cells at an effector-target ratio of 2:1 for 1 day. After that, the supernatant of the co-cultured cells was harvested by centrifugation (1000 rpm, 5 min), and clarified supernatants were aliquoted for subsequent cytokine profiling. The cytokines, including interleukin-2 (IL-2, EHC003.96), interleukin-6 (IL-6, EHC007.96), interleukin-10, (IL-10, EHC009.96), tumor necrosis factor-α (TNF-α, EHC103a.96), interferon-γ (IFN-γ, EHC102g.96), perforin (EHC154.96), granzyme B (EHC117.96) and granulocyte-macrophage stimulating factor (GM-CSF, EHC105.96) were detected by ELISA Kits in accordance with their instructions (Neobioscience Technology, Beijing, China).

### 2.9 In Vivo Anti-Tumor Activity of CAR-T

To directly compare the efficacy of two groups of CAR-T cells in an *in vivo* setting, female NOD-scid mice (3–5 mice per group) were implanted with MDA-MB-231^HER2+^ cells. The mice (6 weeks old, weighing around 18–20 g), supplied by SJA Laboratory Animal Co. (Hunan, China), were housed in pathogen-free facilities. And experimental procedures were approved by the Animal Experimentation Ethics Committee of Yan’an Hospital, Kunming Medical University (No. AEWC-2021100). Briefly, 5 × 10^6^ MDA-MB-231^HER2+^ cells were transplanted into the breast fat pad of each mouse on day-10. On day 0, tumor growth was observed, and then the mammary cancer MDA-MB-231^HER2+^ xenograft mice were randomly divided into control and treatment groups. The mice were injected with saline, trastuzumab (10 mg/kg) and CAR-T cells 2 × 10^7^ cells/100 μL by tail vein on days 0, 7 and 14, respectively. The mice in CAR-T groups were also administered with IL-2 (2000 IU/100 μL each mouse, Peprotech, AF-200-07-250UG, USA) intraperitoneally. Mouse body weights were tracked throughout the study period. At the end of the experiment, the mice were then sacrificed by cervical dislocation and the tumors were weighed and collected for immunofluorescence staining.

### 2.10 Immunofluorescence Detection of Ki-67 and TUNEL in Tumor Tissues

The collected tumor tissue samples from each group were subjected to paraffin embedding. After deparaffinization and antigen retrieval of the tumor tissue paraffin sections, apoptotic cells were detected in 5 μm deparaffinized tumor sections by using CF488 TUNEL Cell Apoptosis Detection Kit (Servicebio, G1504, Wuhan, China) according to the manufacturer’s instructions. Briefly, after sequential PBS rinses, the sections were subjected to enzymatic pretreatment with proteinase K (20 μg/mL) for 20 min at 37°C. Tissue sections were subjected to two 5-min PBS washes under gentle agitation, The slide was first incubated with 50 µL of Equilibration Buffer for 10 min at room temperature, and subsequently incubated with the TUNEL reaction mixture (2 µL recombinant terminal deoxynucleotidyl transferase enzyme, 5 µL CF488-conjugated dUTP, and 50 µL Equilibration Buffer) for 60 min at 37°C in a humidified dark chamber. After incubation, the slides were rinsed with PBS four times. For Ki-67 detection, slides were blocked with 5% BSA in a humidified chamber for 60 min, then incubated with anti-Ki-67 antibody (Proteintech, 27309-1-AP, USA, 1:200) overnight at 4°C. After three PBS washes, FITC-labeled goat anti-rabbit IgG (Thermo Fisher Scientific, 65-6111, Shanghai, China, 1:2000) was dispensed and incubated for 60 min in light-protected conditions. After both detections were washed with PBS, nuclear counterstaining was performed using DAPI (Thermo Fisher Scientific, D1306, China) under light-protected conditions for 10 min. Samples were coverslipped with a rapid-mounting media, and imaged using a fluorescence microscope (Bio-Rad, 742BR1307, USA). Green fluorescence from Ki-67 and TUNEL, respectively, indicated proliferating and apoptotic cells, while DAPI stains the cell nuclei.

## 3 Statistical Analysis

Continuous variables were analyzed using Student’s independent *t*-test or two-way analysis of variance (ANOVA), Tukey’s or Dunnett’s *post hoc* tests for multiple comparisons. A *p*-value < 0.05 is defined as statistically significant. All statistics were performed using GraphPad Prism 8 software (GraphPad Software Inc., San Diego, CA, USA).

## 4 Results

### 4.1 The Optimization of the Ratio of Transposons and Transposases Plasmids for Transfection of Anti-HER2 and Anti-HER2-13 CAR-T Cells

To optimize transfection efficiency, electroporation trials were conducted using three different ratios of transposon to transposase plasmids for the generation of anti-HER2 and anti-HER2-13 CAR-T cells. Flow cytometric assessment showed that the proportion of CD3^+^CAR^+^ cells in anti-HER2 CAR-T populations was highest (53.8%) when a 5 μg:5 μg ratio of transposon to transposase plasmids was used, significantly exceeding the levels observed with 10 μg:5 μg and 10 μg:10 μg ratios (Fig. 1C). Moreover, the number of CD3^+^ CAR^+^ cells in anti-HER2 CAR-T populations was also higher in the group transfected with a 5 μg:5 μg ratio of the transposon to transposase plasmids, when compared with the other two ratio groups (Fig. 1E). Meanwhile, no significant differences in CD3^+^ CAR^+^ expression were observed among anti-HER-13 CAR-T cells transfected with the three different ratios of transposon to transposase plasmids (Fig. 1D). Notably, the anti-HER2-13 CAR-T group transfected with a 10 μg:5 μg ratio of transposon to transposase plasmids exhibited superior proliferation and increased prevalence of CD3^+^CAR^+^ cellular subsets relative to the other ratio groups (Fig. 1E).

### 4.2 No Significant Differences in the Expansion Abilities, CD3^+^CAR^+^ Population, CD4^+^CAR^+^/CD8^+^CAR^+^ Proportions, and Memory and Exhaustion Markers between Anti-HER2 and Anti-HER2-13 CAR-T Cells In Vitro

**Figure 2:**
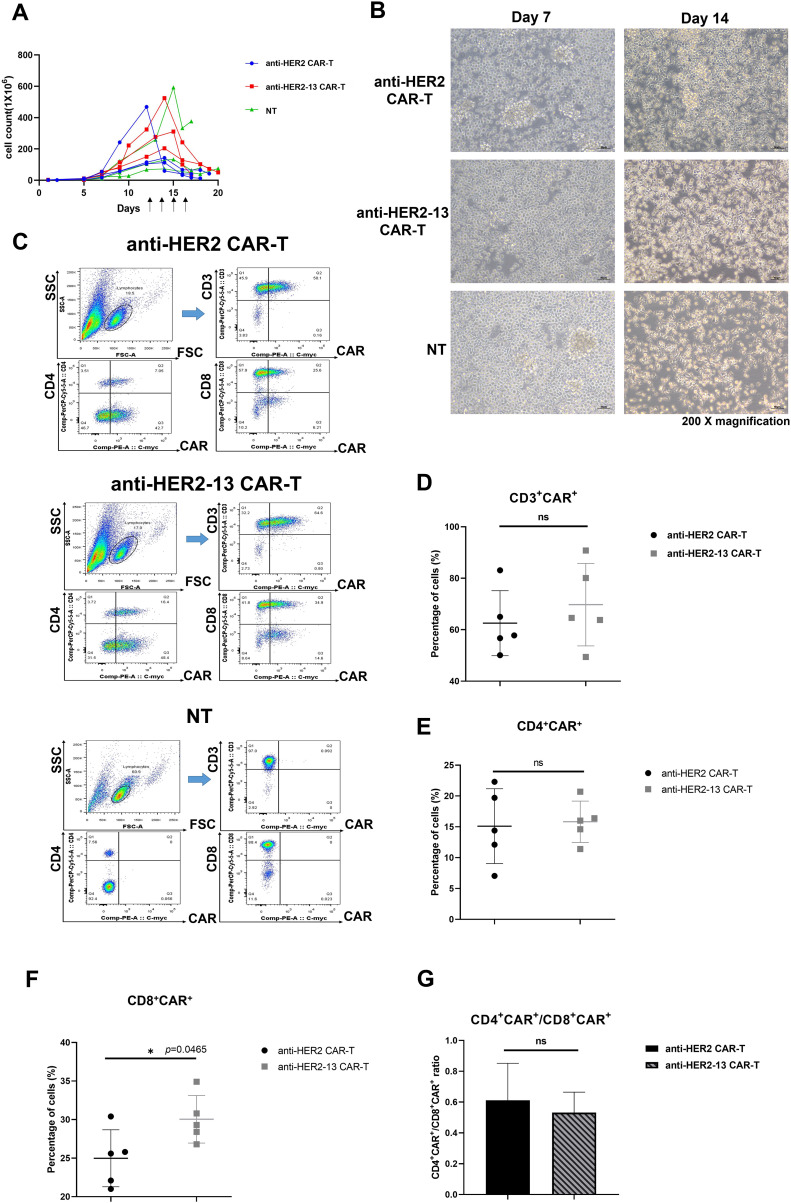
The differences in *in vitro* expansion abilities, CD3^+^CAR^+^, CD4^+^CAR^+^/CD8^+^CAR^+^ expression between anti-HER2 and anti-HER2-13 CAR-T cells. (**A**) The proliferation of CAR-T cells or NT cells which were transfected with empty vector (PB-puro). The arrow indicated the different time points for adding puromycin. The data was from three different donors. (**B**) On the 7th and 14th day, respectively, the representative images of CAR-T cells constructed with two sequences of scFv were taken at a magnification of 200× to compare their morphology. The scale bars in the pictures are 50 μm. (**C**) Representative flow cytometry photos of CD3^+^CAR^+^, CD4^+^CAR^+^ and CD8^+^CAR^+^ expression in the CAR-T cells and NT cells. (**D**–**G**) The analytic results of CD3^+^CAR^+^, CD4^+^CAR^+^, CD8^+^CAR^+^ and CD4^+^CAR^+^/CD8^+^CAR^+^ cells. The data was from five (or seven) different donors. The difference between the anti-HER2 group and anti-HER2-13 group was determined by Student *t*-test, *p < 0.05; ns (not significant, *p* ≥ 0.05), n = 5

As shown in Fig. 2A, we carried out a comparative analysis of the *in vitro* propagation of both CAR-T and NT cells. At days 7 and 14, the morphology of cells were observed under a microscope. The cells displayed a regular, round shape and tended to form clumps. The result demonstrated that CAR-T cells transfected with either of the two scFv sequences exhibited good viability and strong proliferative abilities over time, with no significant differences identified in both CAR-T cell groups (Fig. 2A,B).

To compare the CAR expression between anti-HER2 and anti-HER2-13 CAR-T cells, the transfected cells were cultured for approximately 16–20 days, and subsequently harvested for the detection of CD3^+^CAR^+^, CD4^+^CAR^+^, and CD8^+^CAR^+^ expression using flow cytometry. The percentages of CD3^+^CAR^+^, CD4^+^CAR^+^, and CD8^+^CAR^+^ expression in anti-HER2 CAR-T cells were 59.27 ± 11.84%, 15.11 ± 6.06%, and 24.98 ± 3.69%, respectively. In anti-HER2-13 CAR-T cells, these proportions were slightly elevated, reaching 64.04 ± 16.30% for CD3^+^CAR^+^, 15.82 ± 3.36% for CD4^+^CAR^+^, and 30.04 ± 3.08% for CD8^+^CAR^+^ cells (Fig. 2C–F). Moreover, the CD4^+^CAR^+^/CD8^+^CAR^+^ ratios of anti-HER2 and anti-HER2-13 CAR-T cells were also analyzed. The data revealed that the CD4^+^CAR^+^/CD8^+^CAR^+^ ratio of anti-HER2 CAR-T cells was 0.61 ± 0.24, while anti-HER2-13 CAR-T cells had a ratio of 0.53 ± 0.13. Nevertheless, no statistically significant difference was observed between the two CAR-T cell groups (Fig. 2G). Therefore, aside from the CD8^+^CAR^+^ population, the two CAR-T cell constructs exhibited minimal differences in cell expansion, as well as in the proportions of CD3^+^CAR^+^, CD4^+^CAR^+^ cells in their freshly prepared state.

**Figure 3:**
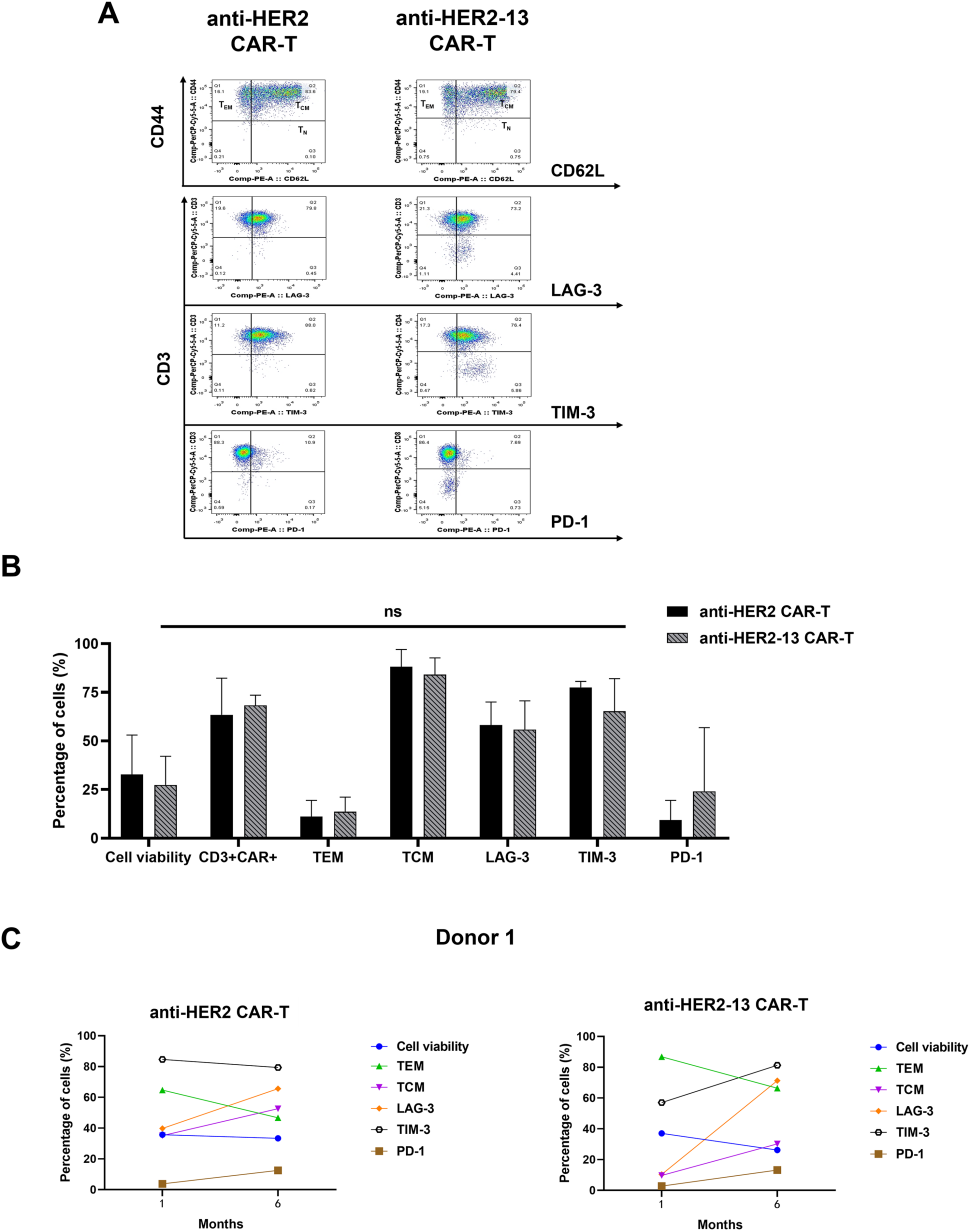

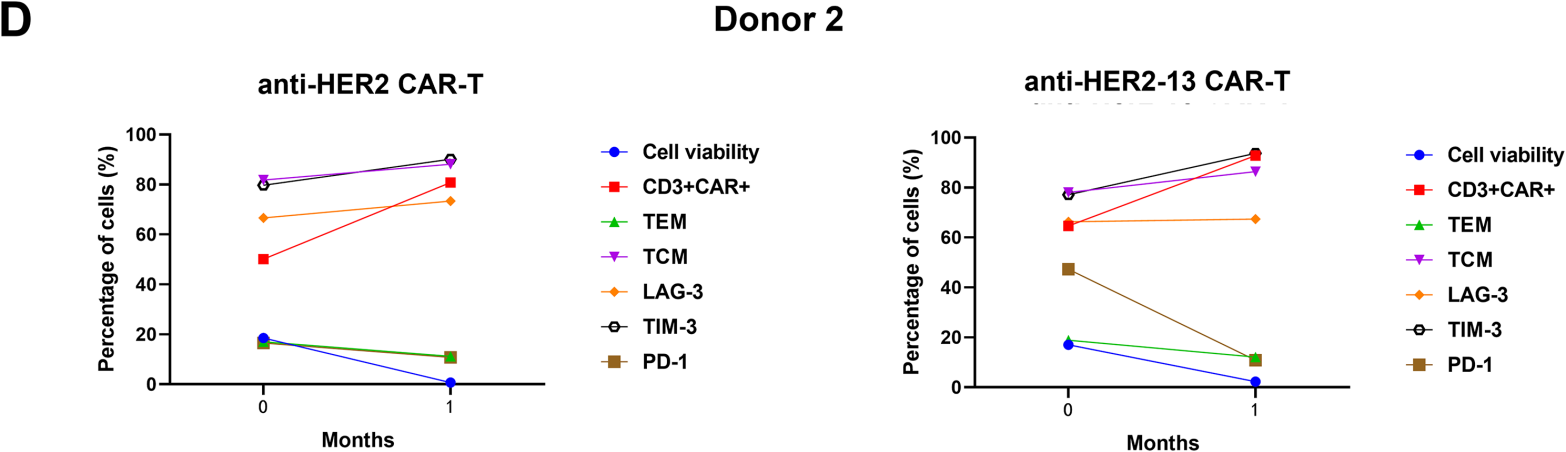
The differences in memory (CD44, CD62L) and characteristic markers of T cell exhaustion (PD-1, LAG-3, TIM-3) between anti-HER2 and anti-HER2-13 CAR-T cells. (**A**) Representative flow cytometry photos of memory and exhaustion marker expression in anti-HER2 and anti-HER2-13 CAR-T cells. (**B**) The analytic results of memory and exhaustion marker expression in CAR-T cell (0: fresh state). The data was from three different donors. (**C**,**D**) The influence on the cell viability, memory (CD44, CD62L) and key exhaustion indicators (LAG-3, TIM-3, PD-1) of anti-HER2 and anti-HER2-13 CAR-T cells under cryopreservation (0: fresh state, 1: one month, 6: six months). The data was from two different donors. The disparity between the anti-HER2 group and the anti-HER2-13 group was assessed through a Student’s *t*-test, which yielded ns (showing non-significant differentials, *p* ≥ 0.05)

CAR-T cells can sustain long-term anti-tumor effects *in vivo* through differentiation into memory subgroups [25]. A previous study has demonstrated that fresh PBMC predominantly adopt a central memory T cell (TCM) phenotype after undergoing cryopreservation [26]. Therefore, the expression of memory-associated and exhausted markers in both fresh and frozen states of both anti-HER2 and anti-HER2-13 CAR-T cells, derived from dual-donor provenance, was analyzed by flow cytometry. Comparative analysis failed to identify any significant disparities between the two CAR-T cell constructs containing different scFvs, derived from the same donor, in terms of effector memory T cell (TEM, CD62L^-^CD44^+^) and central memory T cell (TCM, CD62L^+^CD44^+^) subpopulations, as well as in the expression of immunoexhaustion-associated molecules including PD-1, TIM-3 and LAG-3 (Fig. 3A,B).

In addition, the status of CAR-T cells, including cell viability, expression of exhaustion markers, and the percentages of CAR^+^CD3^+^, TCM, TEM phenotypes, were also evaluated after one-month or six-month cryopreservation. The data showed a decrease in the TEM subpopulation and an increase in the TCM subpopulation in both anti-HER2 and anti-HER2-13 CAR-T cells. At the same time, the expressions of exhaustion markers, such as LAG-3, TIM-3, and PD-1, in both CAR-T groups showed a tendency to increase with prolonged cryopreservation time (Fig. 3C,D).

### 4.3 The Anti-Tumor Efficacy of CAR-T Cells against Various HER2-Positive or HER2-Negative Cancer Cell Lines

**Figure 4:**
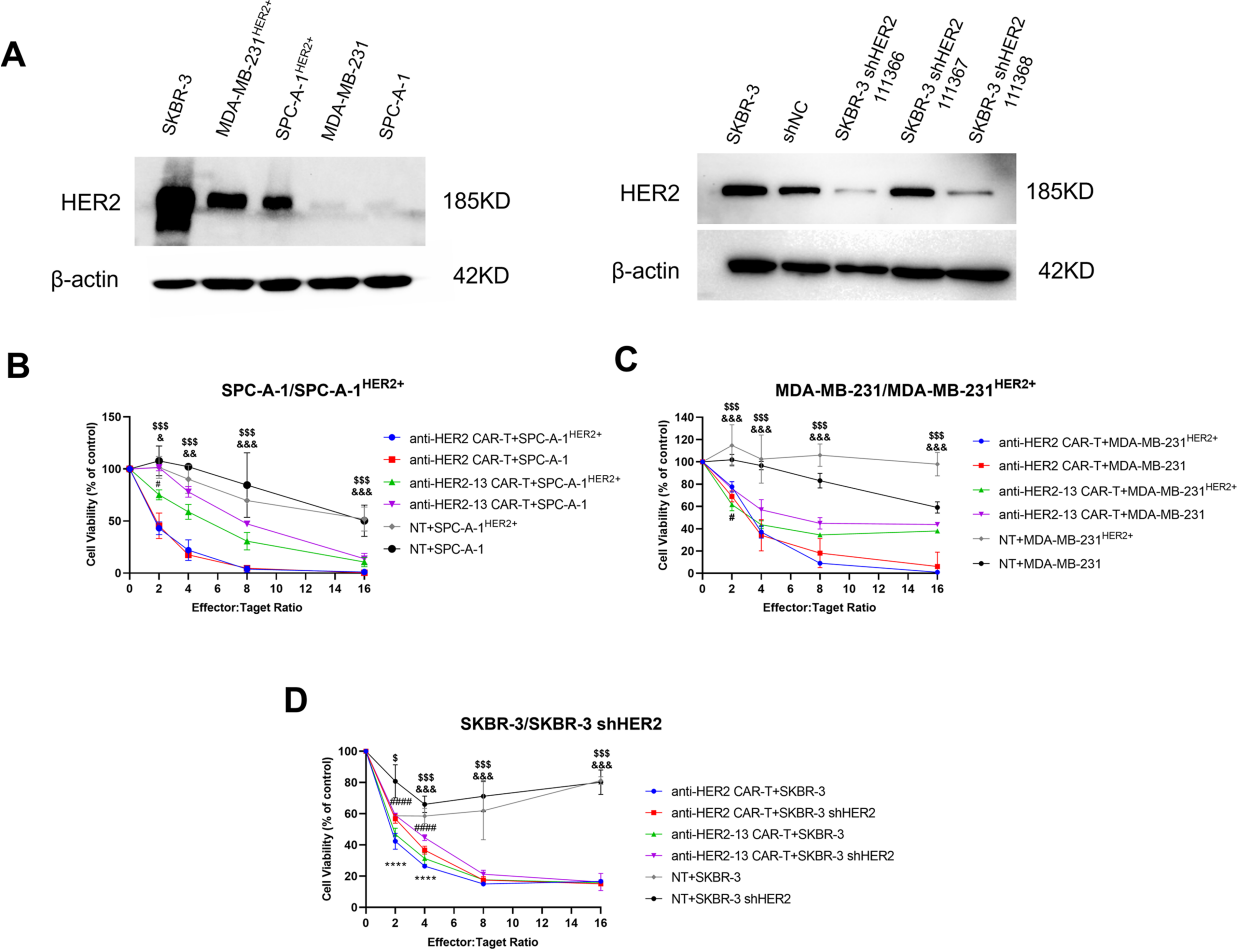
The anti-tumor effects of CAR-T cells on different HER2-positive or HER2-negative cancer cell lines. (**A**) The protein expression levels of HER2 in different tumor cells of lung adenocarcinoma (SPC-A-1^HER2+^/SPC-A-1) and breast cancer (MDA-MB-231^HER2+/^MDA-MB-231 and SKBR-3/SKBR-3 shHER2) were determined by western blotting, and the expression of β-actin was measured to confirm equal protein levels. (**B**–**D**) Cytotoxic effects of CAR-T cells on the above cancer cells. Cell viability was measured by colorimetric CCK-8 assay after treatment with CAR-T cells or NT cells for 24 h. The differences among CAR-T groups and NT on SPC-A-1^HER2+^/MDA-MB-231^HER2+^/SKBR-3 cells were determined by two-way ANOVA and *post hoc* Tukey multiple comparison tests. ^&^*p* < 0.05, ^&&^*p* < 0.01, ^&&&^ *p* < 0.001 anti-HER2-13 CAR-T group vs. NT group; ^$^*p* < 0.05, ^$$$^*p* < 0.001 anti-HER2 CAR-T group vs. NT group. The CAR-T groups on SPC-A-1^HER2+^/SPC-A-1, MDA-MB-231^HER2+^/MDA-MB-231 and SKBR-3/SKBR-3 shHER2 cells were determined by two-way ANOVA and *post hoc* Tukey multiple comparison tests. ^#^*p* < 0.05 anti-HER2-13 CAR-T+SPC-A-1^HER2^ vs. anti-HER2-13 CAR-T+SPC-A-1, and anti-HER2-13 CAR-T+MDA-MB-231^HER2^ vs. anti-HER2-13 CAR-T+MDA-MB-231; ^####^*p* < 0.0001 indicates the statistical differences between anti-HER2-13 CAR-T+SKBR-3 and anti-HER2-13 CAR-T+SKBR-3 shHER2 group; ****p < 0.0001 indicates the statistical differences between anti-HER2 CAR-T+SKBR-3 and anti-HER2 CAR-T+SKBR-3 shHER2 group. The line plots display the mean value ± standard deviation (SD) from biological replicates (n = 3 wells per experiment)

As presented in Fig. 4A, SKBR-3 cells were identified as a type of breast cancer cell line with inherent HER2 overexpression. Nevertheless, HER2 protein expression was markedly reduced in SKBR-3 shHER2 cells relative to their wild-type counterparts. HER2 protein expression was significantly elevated in SPC-A-1^HER2+^ and MDA-MB-231^HER2+^ cells compared to their wild-type counterparts.

Compared with NT cells, both types of CAR-T cells co-cultured with magnetic beads effectively and specifically targeted HER2^+^ tumor cells, including SPC-A-1^HER2+^, MDA-MB-231^HER2+^ and SKBR-3 (Fig. 4B–D). Following 24 h of culture, both types of CAR-T cells significantly induced dose-dependent tumor cell death in all HER2 overexpression cancer cell lines. With an 8:1 ratio of CAR-T cells to target cells, anti-HER2 CAR-T cells exhibited over 80% cytotoxicity against all tested cancer cell lines, regardless of their HER2 expression status, indicating potential off-target effects. This finding indicated that anti-HER2 CAR-T cells possessed strong cytotoxic capabilities; however, their specificity toward HER2-positive tumor cells was limited. Meanwhile, anti-HER2-13 CAR-T cells demonstrated enhanced cytotoxicity toward HER2-positive cells compared with HER2-negative cells, especially at an effector-target ratio of 2:1 on SPC-A-1^HER2+^/SPC-A-1 and MDA-MB-231^HER2+^/MDA-MB-231 cells (*p* < 0.05, Fig. 4B,C). Nevertheless, both anti-HER2 and anti-HER2-13 CAR-T cells exhibited obviously higher tumoricidal effects on SKBR-3 cells compared to SKBR-3 shHER2 cells using a 2:1 or 4:1 effector-to-target cell ratios (*p* < 0.0001, Fig. 4D), likely due to the inherently high and widespread expression of HER2 in the former (Fig. 4A). Despite exhibiting weaker cytotoxicity (~60%) against SPC-A-1^HER2^+^^ and MDA-MB-231^HER2^+^^ cells compared to anti-HER2 CAR-T cells (~80%) at an E:T ratio of 8:1, anti-HER2-13 CAR-T cells may offer superior safety due to their greater specificity for HER2-positive targets.

### 4.4 CAR-T Cell-Mediated Cytokine Induction

**Figure 5:**
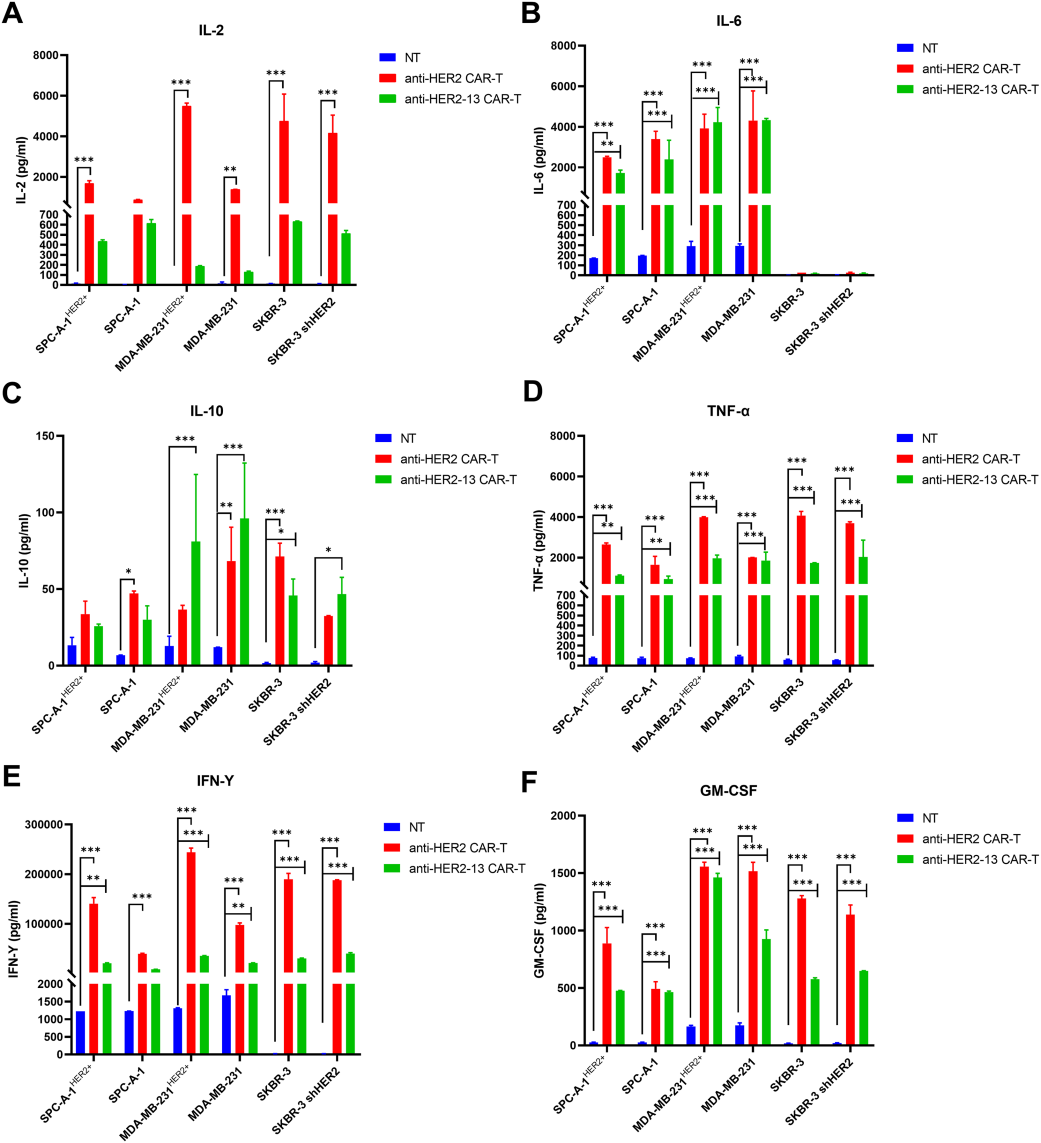

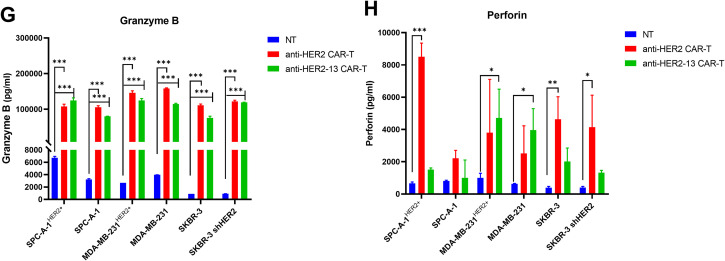
The potential mechanisms involved in cytotoxicities of CAR-T cells against HER2 positive or negative lung adenocarcinoma and breast cancer cell lines. (**A**–**H**) Cytokines (IFN-γ, TNF-α, IL-10, IL-6, IL-2, GM-CSF) and effector molecule (perforin, granzyme B) production were quantified by ELISA following CAR-T cell co-culture with tumor targets at a 2:1 effector-to-target ratio. Compared with the NT control group, the statistical significance was assessed using two-way ANOVA followed by Dunnett’s post hoc test: *p < 0.05, **p < 0.01, ***p < 0.001; the data were from two independent experiments with three wells each

To further investigate the anti-tumor efficacy of CAR-T cells and understand the underlying mechanisms, we performed ELISA experiments to measure the release of key cytokines, including IL-2, IL-6, IL-10, TNF-α, GM-CSF, perforin, and granzyme B from the supernatant of CAR-T cells and NT cells co-cultured with tumor cells at an E:T ratio of 2:1 for 24 h. Our results demonstrated that the differences in cytokine secretion among the two CAR-T cell types and NT cells co-cultured with tumor cells were in line with their respective cytotoxic effects against tumor cells. Specifically, both anti-HER2 and anti-HER2-13 CAR-T cells exhibited significantly elevated secretion of IL-2, IL-6, TNF-α, IFN-γ, and granzyme B relative to NT cells under 2:1 effector-to-target conditions (Fig. 5A,B,D,E,G). These data collectively imply that these cytokines play a crucial role in mediating the tumoricidal efficacy of CAR-T cells. Furthermore, both CAR-T groups also produced higher levels of IL-10, perforin, and GM-CSF by contrast to NT cells (Fig. 5C,F,H). The increased production of these factors by CAR-T cells indicated their possible involvement in immune regulation and enhancing overall tumor elimination.

### 4.5 Anti-Tumor Capacity of HER2-Targeted CAR-T Cells with Different scFv in the Xenograft Model

**Figure 6:**
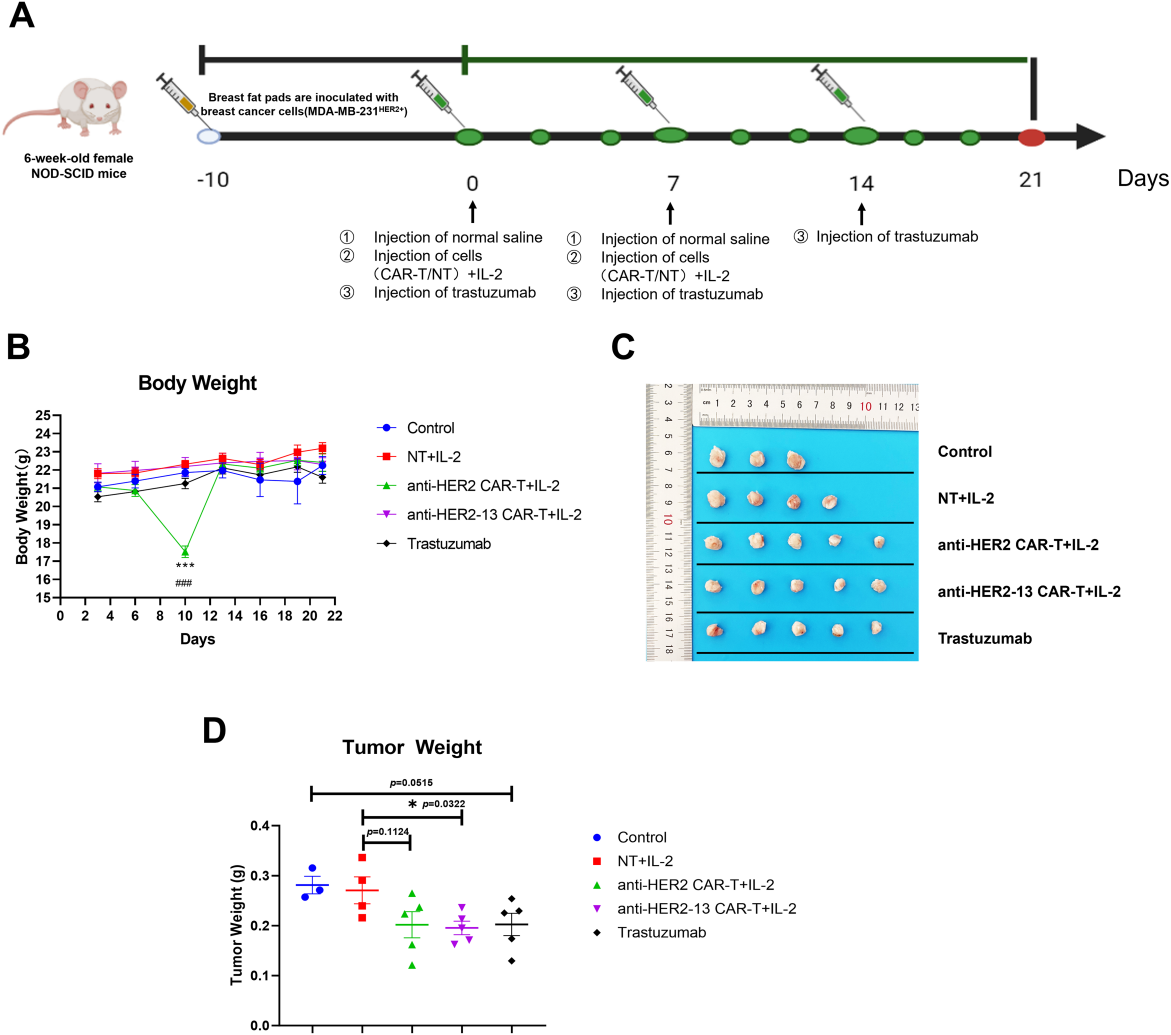
The effects of anti-HER2, anti-HER2-13 CAR-T cells and trastuzumab on the MDA-MB-231^HER2+^ xenograft mouse model. (**A**) The establishment and treatment pattern of the breast cancer MDA-MB-231^HER2+^ mouse model. (**B**) The body weight of mice in each group was measured throughout the entire treatment period. (**C**) The representative photographic documentation of resected murine mammary tumors. (**D**) The tumor weight of dissected MDA-MB-231^HER2+^ in mean ± SEM of 3–5 tumors. ^###^*p* < 0.001 anti-HER2 CAR-T+IL-2 group vs. anti-HER2-13 CAR-T+IL-2 group, ***p < 0.001, NT+IL-2 group vs. anti-HER2 CAR-T+IL-2 groups, by two-way ANOVA followed by *post-hoc* Tukey’s multiple comparison. *p < 0.05, NT+IL-2 group vs. anti-HER2-13 CAR-T+IL-2 group by Student *t*-test; n = 5 per group

Based on the promising results observed in *in vitro* studies, additional *in vivo* investigations were performed employing a xenograft tumor model (Fig. 6A). Our result demonstrated that the Trastuzumab group had a certain therapeutic effect relative to the control group, although it was not statistically significant (*p* = 0.0515, Fig. 6D). This result confirmed the successful establishment of our animal model, enabling the evaluation of different treatment modalities. Given that previous studies have demonstrated the beneficial effects of IL-2 on CAR-T cells or T cells [27,28], IL-2 was co-administered with CAR-T cells in the murine model. The data demonstrated that both CAR-T cell groups with IL-2 injection exhibited anti-tumor effects compared to the NT+IL-2 group, with the anti-HER2-13 CAR-T group showing a statistically significantly greater reduction in tumor weight (*p* = 0.0322) (Fig. 6C,D). Moreover, when examining the overall changes in body weight among the different treatment groups, we observed a temporary decrease in murine body weight in the anti-HER2 CAR-T treatment group. However, no statistically significant alterations in body weight were observed in the other treatment groups throughout the entire treatment period (Fig. 6B).

**Figure 7:**
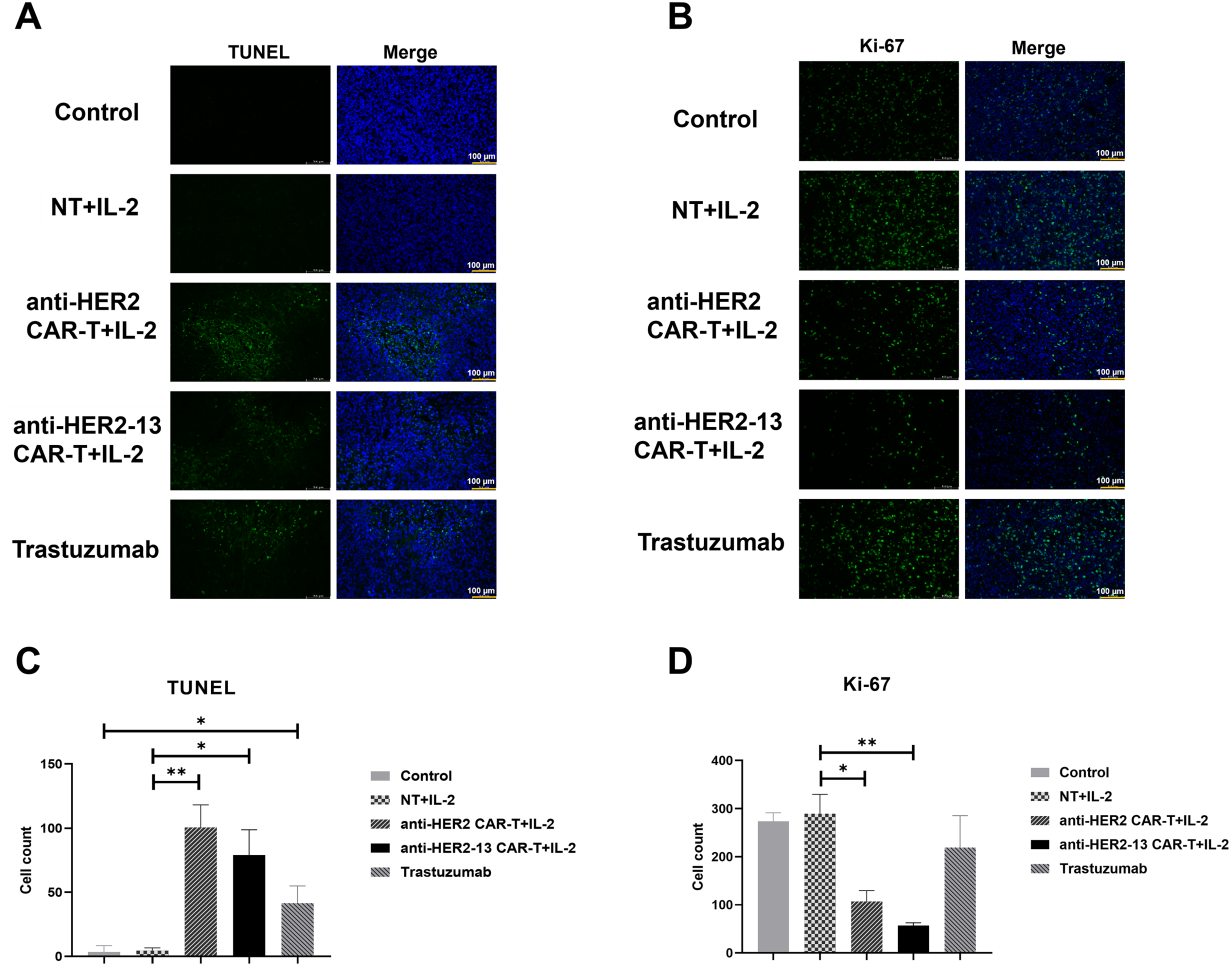
Tumor sections from each group were subjected to immunofluorescence staining. (**A**,**B**) The representative images of TUNEL (apoptosis detection) and Ki-67 (proliferation) on the panel. The scale size is 200x and scale bars in the pictures are 100 μm. (**C,D**) The histograms on the panel showed quantified results of positively stained cells. Statistical comparisons were performed using two-way ANOVA followed by Tukey-Kramer post hoc tests *p < 0.05; **p < 0.01; n = 3–5. Triplicate images were randomly acquired for every tissue section

At the study endpoint, Ki-67 and TUNEL immunofluorescence staining were used to detect proliferating and apoptotic cells, respectively, in tumor tissue. The findings revealed a statistically significant rise in the frequency of apoptotic cells in the Trastuzumab group in comparison with the control group (Fig. 7C, *p* < 0.05). Furthermore, both CAR-T cell treatment groups exhibited a significantly higher number of apoptotic cells as opposed to the NT group (Fig. 7A,C), indicating that CAR-T cells could effectively induce tumor cell apoptosis and thereby exert their anti-tumor effects. The results of the Ki-67 assay showed that no significant differences in Ki-67 expression in tumor tissues among the Trastuzumab-treated group, the NT cell-treated group, and the control group. However, both CAR-T cell treatment groups exhibited statistically significant reductions in Ki-67 expression compared to the NT cells treatment group. This suggested that both CAR-T cells effectively suppressed the proliferative capacity of tumor cells (*p* < 0.05, *p* < 0.01), with the anti-HER2-13 CAR-T treatment group exhibiting a notably stronger effect (Fig. 7B,D).

## 5 Discussion

The current approach for CAR-T cell transfection primarily relies on viral vectors. However, their high immunogenicity and limited cargo capacity restrict their further clinical application. Given the advantages of non-viral vectors, including low immunogenicity, high safety and cost-effectiveness [29], we developed an optimized *PiggyBac* (PB) transposon-based platform for genetic modification, delivering cDNA to express HER2-CD28-4-1BB-CD3ζ CAR (Fig. 1A). Previous studies have demonstrated that varying ratio of transposon to transposase plasmids influences cell transfection efficiency [21,30]. Although HER2-targeting CAR-T cells generated using *PiggyBac* transposon system have been successfully studied [22], the optimal transposon-to-transposase plasmids ratios for different HER2-targeting CAR constructs remain unexplored. Furthermore, some tumors express HER2 at levels that are insufficiently recognized by trastuzumab compared to normal tissue, potentially leading to off-target effects. Therefore, our study utilized a novel sequence of a humanized mAb against HER2 to conduct CAR structure, aiming to reduce the off-target effect. According to the HADDOCK docking results, the humanized anti-HER2-13 scFv exhibited a stronger predicted binding affinity to the HER2 antigen, with a HADDOCK score of –151.3 ± 10.9, compared to –143.1 ± 1.7 for the original anti-HER2 scFv. And HER2-13 scFv engages more interfacial residues than HER2 scFv in the simulated binding. (Supplementary Fig. S1). Moreover, some studies have suggested that the optimal ratio of transposon to transposase plasmids is 2:1, and exceeding this ratio led to inhibition of transfection efficiency as well as cell proliferation [21]. Additionally, another study reported that a 1:1 ratio of transposon to transposase plasmids yielded more favorable results [31]. Our results showed that the optimal transposon and transposase plasmids ratios were differed between anti-HER2 and anti-HER2-13 CAR-T cells, although the underlying reason remains to be further investigated. The observed difference in ratio requirements may stem from the distinct scFv sequences of the anti-HER2 and anti-HER2-13 CAR constructs, which could influence transcriptional or translational efficiency, or alter mRNA stability [32].

Since FDA-cleared CAR-T therapeutics are produced in centralized manufacturing facilities, cryopreservation is required to enable the transport of the final cell product back to the patient. Thereby, the impact of cryopreservation on CAR-T cell functionality is a critical factor influencing both production processes and therapeutic efficacy. In view of this, we evaluated cell viability, phenotypic characteristics—including TEM and TCM subpopulations—and the expression of exhaustion markers in our CAR-T cells. The data showed a decrease in TEM cells accompanied by an increase in TCM cells in both cryopreserved CAR-T cell groups derived from two donors. Notably, both anti-HER2 and anti-HER2-13 CAR-T cells exhibited similar trend (Fig. 3C,D). We speculate that TEM subpopulation cells were more sensitive to cryopreservation or temperature fluctuations. Notably, although the proportion of CD3^+^CAR^+^ cells remained relatively stable or even slightly increased, cell viability decreased over time. In addition, the expression level of exhaustion markers—LAG-3, TIM-3 and PD-1—in both CAR-T cell groups tended to increase with the prolonged cryopreservation. These findings suggest that extended cryopreservation may negatively impact the functional efficacy of CAR-T cells to some extent. This adverse effect can be effectively mitigated through optimized cryopreservation protocols. For instance, novel cryoprotectants have been employed as alternatives to conventional dimethyl sulfoxide (DMSO), minimizing cellular damage [33]. Additionally, controlled-rate freezing protocols have been implemented to minimize ice crystal-induced cellular damage [34].

Several clinical or pre-clinical studies had illustrated that the administration of IL-2 following adoptive transfer cells therapies could enhance the persistence and expansion of the transferred cells [35–37]. Therefore, all mice in the present study received intraperitoneal IL-2 following CAR-T infusion. Actually, in a preliminary experiment, CAR-T cells were administered without IL-2 support, and neither of the two CAR-T groups exhibited anti-tumor activity (data not shown), suggesting that IL-2 indeed serves a pivotal role in enhancing the *in vivo* survival and functionality of CAR-T cells [38]. This experiment underscores the critical role of IL-2 infusion in enabling CAR-T cells to achieve potent therapeutic efficacy *in vivo*. However, the clinical feasibility of exogenous IL-2 dependency has raised significant concerns. To address this, strategies such as optimized cytokine cocktails, co-administration of IL-15, or engineering autocrine IL-15 secretion into CAR-T cells have been proposed to reduce reliance on exogenous IL-2 [39,40]. In addition, we observed a transient decrease in body weight in mice treated with anti-HER2 CAR-T cells following the second CAR-T cell injection. Although the mice recovered by day 13, this observation may raise concerns regarding potential toxicity, possibly due to off-target effects. The observation that body weight remained stable in anti-HER2-13-treated mice implies this construct may have a more favorable safety profile with less off-target toxicity than its anti-HER2 counterpart. However, because the effect was temporary, no additional pathological data was collected. Meanwhile this study utilized immunodeficient NOD-scid mouse model, it should be noted that these systems cannot fully recapitulate the authentic biological behavior of CAR-T cells in human patients. A notable limitation of this study is the absence of experimental validation of the antibody–antigen interactions. The binding affinity and interface were assessed exclusively through *in silico* docking analyses, which may not fully capture the complexity of biological interactions. Future studies should incorporate biophysical or biochemical assays to experimentally confirm the predicted binding interactions. Our animal studies showed that anti-HER2-13 CAR-T cells performed equivalently or potentially better than trastuzumab (Fig. 6). While demonstrating comparable short-term efficacy, CAR-T therapy offers a distinct advantage in therapeutic persistence. Unlike antibody-drug conjugates such as trastuzumab emtansine (T-DM1) that require repeated dosing, CAR-T cell therapies demonstrate sustained persistence and establish immunological memory [41,42]. This sustained activity may provide critical protection against tumor recurrence. Verification of this hypothesis will require extended longitudinal studies with prolonged observation periods. Furthermore, the anti-tumor efficacy could also potentially be enhanced through localized CAR-T cell delivery or combinatorial approaches integrating CAR-T cells with monoclonal antibodies [43,44].

Taken together, we successfully developed the HER2-specific CAR-T cells incorporating novel sequences of scFv using *PiggyBac* transposon vector via electroporation. These CAR-T cells demonstrated potent tumor-suppressive capacity *in vitro* and *in vivo*. Notably, the anti-HER2-13 CAR-T cells exhibited improved safety profiles, likely due to their enhanced specificity for HER2-positive cells. These results highlight the clinical potential of HER2-directed CAR-T cell therapies, particularly those employing the anti-HER2-13 scFv.

## Supplementary Materials



## Data Availability

Not applicable.

## Supplementary Materials

The supplementary material is available online at https://www.techscience.com/doi/10.32604/or.2025.065394/s1.
