# Supplementary material for: Investigation on the Anti-Cancer Effects of HER2-Targeted CAR-T Cells Engineered Using the PiggyBac Transposon System
Source: Oncol Res. 2025 Oct 22;33(11):3447–67. doi: 10.32604/or.2025.065394 (PMC12573187; doi:10.32604/or.2025.065394)
Supplement: Supplementary file 1 [file OncolRes-33-65394-s001.docx]

Supplementary Table S1. Sequence of the anti-HER2 single-chain antibody gene.

| **scFv** | **gene sequence** |
| --- | --- |
| HER2 | 5’-ATGGATTTCCAGGTGCAGATATTCTCCTTTCTCCTCATATCAGCCTCTGTGATCATGAGCAGAGGAGATATACAGATGACACAATCTCCATCTAGTCTGTCTGCCTCAGTCGGTGATCGCGTTACCATCACTTGTAGGGCAAGCCAGGACGTGAATACAGCCGTTGCCTGGTATCAGCAGAAACCTGGAAAGGCTCCCAAGCTGCTGATCTATAGCGCCAGTTTCCTGTATAGCGGAGTTCCCTCCAGATTCAGTGGTAGCAGGAGTGGCACAGATTTCACTCTCACAATCAGCAGCCTCCAGCCAGAGGACTTTGCTACTTACTATTGCCAACAGCACTATACCACTCCTCCCACATTTGGCCAGGGCACCAAAGTCGAGATTAAGCGCACAGGGTCTACAAGCGGTAGCGGAAAGCCAGGATCAGGCGAAGGCAGCGAGGTCCAGCTGGTGGAATCTGGAGGTGGACTGGTGCAACCCGGAGGATCTCTGCGCCTCTCATGTGCCGCAAGCGGGTTCAACATTAAGGACACTTACATTCACTGGGTCAGGCAGGCACCTGGGAAGGGACTCGAATGGGTGGCTAGGATCTATCCAACCAACGGCTACACTCGCTACGCAGACTCAGTCAAGGGTCGCTTTACCATATCAGCCGATACTTCTAAGAACACCGCCTACCTGCAAATGAACTCACTGAGGGCTGAGGACACCGCAGTGTACTACTGCTCTAGGTGGGGTGGAGATGGCTTCTATGCTATGGATGTGTGGGGGCAGGGCACCCTCGTGACCGTCAGTAGTGCCGCTGGGTCA-3’ |
| HER2-13 | 5’- ATGGCACAGGTACAGCTGCAGCAGTCAGGAACTGAAGTGGTAAAGCCTGGGGCTTCAGTGAAGTTGTCCTGCAAGGCTTCTGGCTACATCTTCACAAGTTATGATATAGACTGGGTGAGGCAGACGCCTGAACAGGGACTTGAGTGGATTGGATGGATTTTTCCTGGAGAGGGGAGTACTGAATACAATGAGAAGTTCAAGGGCAGGGCCACACTGAGTGTAGACAAGTCCTCCAGCACAGCCTATATGGAGCTCACTAGGCTGACATCTGAGGACTCTGCTGTCTATTTCTGTGCTAGAGGGGACTACTATAGGCGCTACTTTGACTTGTGGGGCCAAGGCACCCTGGTCACCGTCTCCTCAGGCGGCGGCGGCTCTGGCGGAGGTGGCAGCGGCGGTGGCGGATCCGACATCGTGATGACCCAGTCTCCATCCTCCCTGTCCGCATCTGTTGGAGACAGAGTCTCCATCACTTGCCGGGCAAGTCAGAACATTGGCCAGCGTTTGAATTGGTATCAGCAGCAACCAGGGAAAGCCCCTAAAGTCCTGATCTATGCTACATCCAAATTGCACATTGGGGTCCCATCAAGATTCAGTGCCAGTGGATCTGGGACAGAATTCAGTCTCACCATCAGCGCTCTGCAACCTGAAGATTTTGCAACTTATTATTGTCAACAGAGCTACAGTCTCCCGCTCGCTTTCGGCGGAGGGACCAAGCTGGAGATCAAACGT-3’ |

HER2: anti-HER2 sequence (trastuzumab); HER2-13: anti-HER2-13 sequence (from combinatorial cellular library of CARs)


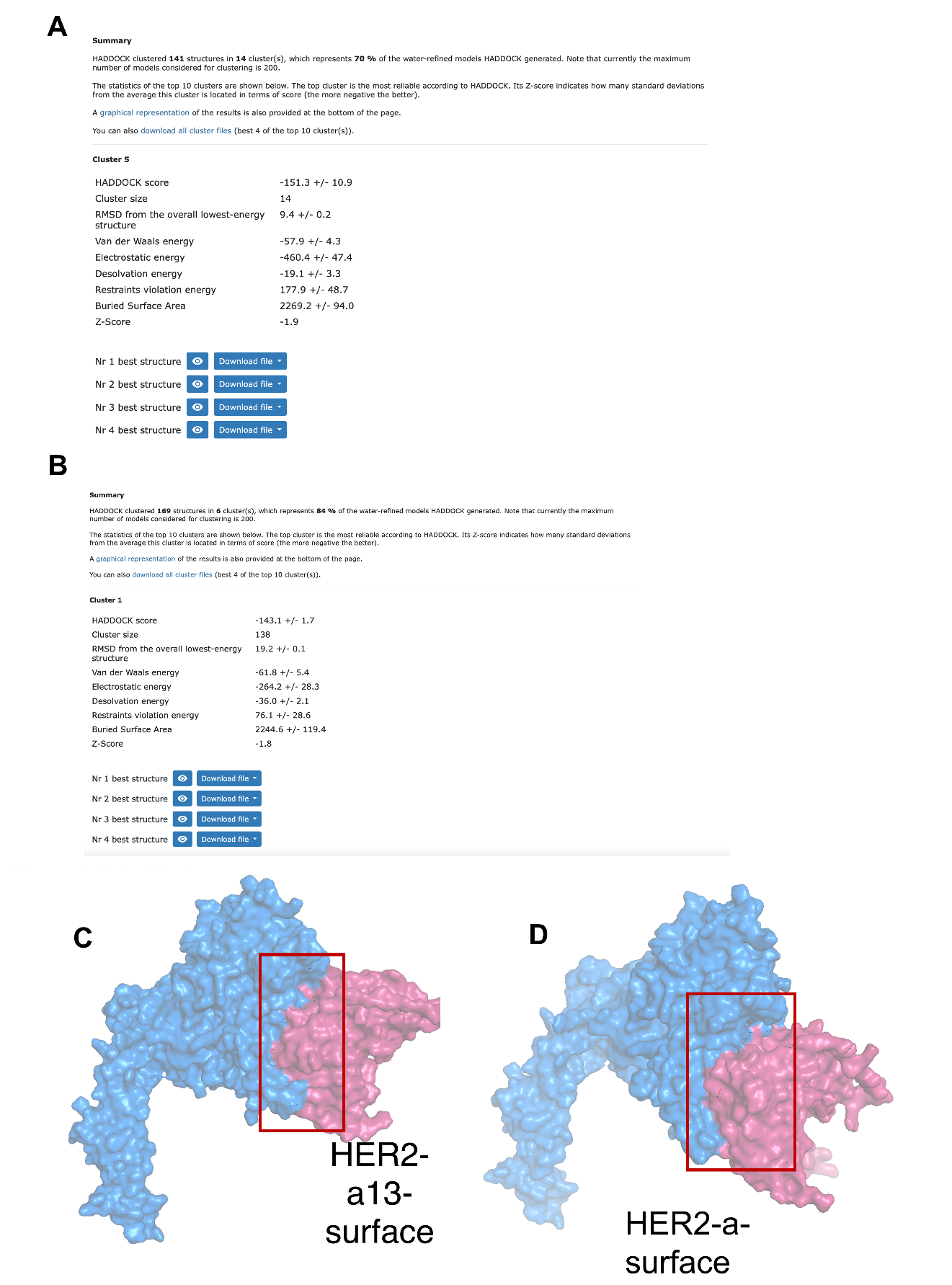


Supplementary Figure S1. The result of the binding score and residues participating in the modeled binding between the antigen and humanized anti-HER2-13 or anti-HER2 scFv (A) HADDOCK score for anti-HER2-13 scFv. (B) HADDOCK score for anti-HER2 scFv. (C&D) The HER2 antigen in complex with two scFvs was visualized using PyMOL, and their binding interfaces were analyzed. The amino acid residues involved in the modeled binding interface were highlighted with black dashed lines.
